# Myocardial perfusion recovery induced by an α-calcitonin gene-related peptide analogue

**DOI:** 10.1007/s12350-021-02678-8

**Published:** 2021-06-04

**Authors:** Simon Bentsen, Anette Sams, Philip Hasbak, Lars Edvinsson, Andreas Kjaer, Rasmus S. Ripa

**Affiliations:** 1 Department of Clinical Physiology, Nuclear Medicine & PET and Cluster for Molecular Imaging, Rigshospitalet and University of Copenhagen, Copenhagen, Denmark; 2 Department of Clinical Experimental Research, Glostrup Research Institute, Glostrup University Hospital, Nordstjernevej 42, 2600 Glostrup, Denmark

**Keywords:** Physiology of myocardial/coronary perfusion, Myocardial ischemia and infarction, SPECT, MPI

## Abstract

**Background:**

Endogenous calcitonin gene-related peptide (CGRP) induces cardioprotective effects through coronary vasodilation. However, the systemic administration of CGRP induces peripheral vasodilation and positive chronotropic and inotropic effects. This study aims to examine the net effect on coronary perfusion of the systemically administered α-calcitonin gene-related peptide analogue, SAX, in rats during myocardial infarction.

**Methods:**

Forty Sprague-Dawley rats underwent myocardial infarction. Following left anterior descending artery occlusion, [^99m^Tc]Tc-sestamibi was administered to determine the myocardial perfusion before treatment. Twenty minutes, 24 and 48 h after [^99m^Tc]Tc-sestamibi injection, the rats were treated with either SAX or placebo. Final infarct size was determined three weeks later by [^99m^Tc]Tc-sestamibi SPECT/CT scan.

**Results:**

Thirty-one rats survived the surgery and 20 completed the follow-up SPECT/CT scan (SAX n = 12; Placebo n = 8). At baseline, there was no difference in size of perfusion defect between the groups (*P* = .88), but at follow-up the SAX group had improved myocardial recovery compared to the placebo group (*P* = .04), corresponding to a relative perfusion recovery of 55% in SAX-treated rats.

**Conclusion:**

The CGRP analogue, SAX, has a cardioprotective effect in this rat model of myocardial infarction, improving myocardial perfusion recovery after chronic occlusion of the coronary artery.

**Supplementary Information:**

The online version contains supplementary material available at 10.1007/s12350-021-02678-8.

## Introduction

Myocardial infarction (MI) is the leading cause of heart failure, despite the introduction of primary percutaneous coronary intervention (PCI).^1^ PCI has improved survival after an MI, but the recovering myocardium is still impaired after PCI. Therefore, it is crucial to develop new treatments to protect and improve myocardial recovery after an MI.

After the onset of an MI, there is insufficient oxygen supply to the myocytes. To protect the myocytes prior to PCI, it is crucial to reduce cardiac workload. Vasodilation of the coronary arteries, the peripheral arterial bed, and venous capacitance vessels is a known and used method of reducing afterload and the myocardial workload.^2^ However, peripheral vasodilation can increase the workload of the heart because of compensatory elevated heart rate, which could lead to increased ischemic damage. Calcitonin gene-related peptide (CGRP) is one of the most potent vasodilators known.^3^,^4^ The vasodilatory actions of CGRP are through CGRP receptors, which comprise calcitonin receptor-like receptor (CRLR) and receptor activity-modifying protein 1 (RAMP1).^5^ These receptors are present in smooth muscle and in endothelium, and although the main vascular effect is endothelium-independent vasodilation in the intramural coronary arteries,^6^ CGRP has also been shown to promote other effects, e.g., endothelial proliferation and angiogenesis.^7^

Endogenous CGRP has been associated with preconditioning-induced cardio protection during experiments using myocardial infarct reperfusion models.^8^,^9^ Importantly, exogenous CGRP has been shown to induce similar cardioprotective effects in isolated hearts and CGRP antagonism reduces the cardioprotective effects.^4^ Also, exogenous CGRP induces positive chronotropic and inotropic effects on isolated guinea pig hearts and isolated human myocardial trabeculae.^10^ In three clinical studies with CGRP infusion for the treatment of cerebral vasospasm, adverse effects of increased heart rate and peripheral hypotension were observed, and the studies were stopped before completion.^11^-^13^

Recently, a metabolically stable CGRP analogue, SAX, with a lipophilic tail has been synthesized.^14^ This analogue reverses hypertension and complications of hypertension in experimental animal studies.^15^ Data show that SAX and CGRP display similar pharmacological actions, but the potency of peripheral vasodilation induced by SAX is approximately 10 times lower than native CGRP.^14^-^16^ The half-life and the molecular size of SAX are increased, as compared to those of CGRP.^14^

The aim of this study was to examine a potential cardioprotective effect of SAX in rats undergoing experimental acute myocardial infarction by permanent left anterior descending (LAD) occlusion. We hypothesized that SAX will increase perfusion of the acutely hypoperfused area without promoting increased workload on the heart and that this will cause improved recovery of the myocardial perfusion.

## Materials and Methods

### Ethical Statement

The Danish Animal Experiment Inspectorate approved experimental protocols (Permit No. 2016-15-0201-00920). All animal procedures performed are in accordance with the guidelines in Directive 2010/63/EU of the European Parliament on the protection of animals used for scientific purposes.

### Experimental Animals

Outbred male Sprague-Dawley rats were used for the animal study (genotype Tyrc/Tyrc, strain RjHan:Sd, Janvier, France). The rats were six weeks old on arrival at our facility and were acclimatized for 7-12 days before entering the study. All animals were cared for at an on-site core animal facility. The animals were housed in IVC Greenline Double Decker cage (Tecniplast group, Italy); each cage with three rats. The cages were kept at 21 ± 2 °C with a 12:12-h dark:light cycle. The animals had access to water and standard rodent chow ad libitum.

### Study Design

**Figure 1:**
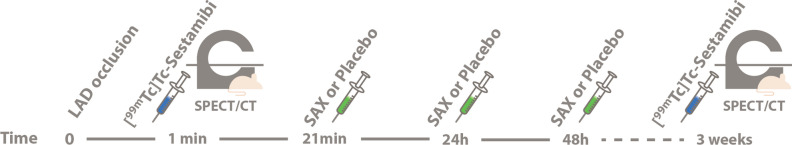
Workflow of this study: First, the rats underwent LAD occlusion. After one minute, [^99m^Tc]Tc-sestamibi was injected for SPECT/CT scan. Twenty minutes later, the rats were treated with either SAX or placebo. The rats were again treated with SAX or placebo at 24 and 48 h after LAD occlusion. Three weeks after a [^99m^Tc]Tc-sestamibi follow-up, SPECT/CT was performed

The study was designed to determine the effect of SAX on myocardial perfusion recovery when SAX was administered after experimental coronary occlusion. Forty rats were included, and open-chest LAD ligation induced an experimental myocardial infarction. The rats were randomly assigned to either SAX or placebo treatment. One minute after confirmed LAD occlusion, the rats were administered [^99m^Tc]Tc-sestamibi to determine the size of the acute myocardial perfusion defect with single positron emission computed tomography (SPECT/CT). Twenty minutes after [^99m^Tc]Tc-sestamibi injection, the rats were intraperitoneally injected with SAX (100 nmol/kg) or placebo. The SAX or placebo treatment was repeated at 24 and 48 h after surgery subcutaneously. Three weeks after myocardial infarction, the final infarct size was determined by [^99m^Tc]Tc-sestamibi SPECT/CT (Figure 1). The sedated rats were euthanized by method of cervical dislocation immediately after follow-up SPECT/CT.

### Drug Formulation and Vehicle

The CGRP analogue, SAX, was synthesized (> 84% purity), stored, and formulated (50 nmol/ml vehicle) as previously described.^14^ The vehicle (.2 M Mannitol, 5% Cyclodextrin, 1.6% ammonium acetate in an aqueous solution at pH 6.5) without SAX was used for placebo treatment. The formulations were stored at 4 °C.

### Experimental Procedures

#### Coronary Occlusion

The rats were anesthetized with 4% sevoflurane and then intubated and ventilated (UNO micor-ventilator-O3, the Netherlands) and kept anesthetized with sevoflurane 2.5% to 4%. The rats were placed on a heated surface during surgery. A 24G intravenous catheter was placed into the tail vein (Vasofix^®^ Safety, Braun, Denmark). Before surgery, the rats were treated subcutaneously with buprenorphine .05 ml/kg. After surgery, the rats were treated four times per day for the first 24 h, and three times per day for the next 72 h, with buprenorphine.

The chest of the animals was shaved, and iodine was used for sterilization. After ensuring sterile surgical conditions, an incision was made in the skin and a thoracotomy was performed at the third or fourth intercostal space, depending on size of the rat. The pericardium was identified and opened. The LAD was identified and permanently ligated 5 mm distal to branching off the left coronary artery with a 5-0 polypropylene suture. The myocardium was observed for ischemia. If there was no discoloration of the myocardium, another suture of the LAD was made until discoloration of the myocardium confirmed ischemia. One minute after successful ligation, the rats were injected intravenously with [^99m^Tc]Tc-sestamibi. After injection of [^99m^Tc]Tc-sestamibi, suture type vicryl 4-0 was used to close the thoracotomy, muscle layer, and skin.

#### Pharmaceutical Treatment

The rats were injected with either 100 nmol/kg SAX or a similar volume of placebo. Three dosages were administered. First dosage was administered intraperitoneally 21 min after LAD ligation. This method ensures that the injection of SAX or placebo was administered 20 min after injections with [^99m^Tc]Tc-sestamibi. This time window allowed the [^99m^Tc]Tc-sestamibi to be trapped in the myocytes before any potential influence of the SAX or placebo. Two additional SAX or placebo doses were given subcutaneously, at 24 and 48 h after myocardial infarction.

#### In Vivo SPECT/CT Imaging

The rats underwent acute SPECT/CT scan one hour after surgery (to determine baseline non-perfused area). In Sprague-Dawley rats, [^99m^Tc]Tc-sestamibi clears from the blood rapidly and is taken up in the heart with very little redistribution.^17^ Therefore, the injection of [^99m^Tc]Tc-sestamibi 20 min before injection of SAX or placebo enables the detection of the non-perfused area before any potential impact of the first SAX injection.

Three weeks after surgery, the rats were follow-up scanned with SPECT/CT. This enabled a determination of final infarct size.^18^ All imaging was performed on a preclinical SPECT/CT scanner (Mediso Nanoscan, Hungary).

The rats were anesthetized with sevoflurane 4% for the SPECT/CT scan. On the day of the surgery, the rats were perioperatively injected with [^99m^Tc]Tc-sestamibi (median 106 mBq[99; 116]), as described above. Twenty-one days after surgery, the rats were anesthetized with sevoflurane 4%. A 24G intravenous catheter was placed in the tail vein (Vasofix^®^ Safety, Braun, Denmark). The rats were then injected with [^99m^Tc]Tc-sestamibi (median 122 [106; 129]).

During the SPECT/CT scan, the rats were placed on a special bed, provided by the vendor, in a prone position. The rats were monitored by way of electrocardiography (ECG), respiratory rate, and core temperature. A computed tomography (CT) scout image was obtained to ensure correct positioning of the SPECT detector’s field of view over the heart. One hour after injection of [^99m^Tc]Tc-sestamibi, the SPECT acquisition was initiated. Scan time was between 20 and 40 min, depending on injected activity, adjusted to ensure a number of counts above 100,000 counts/frame/detector. For attenuation correction and anatomical co-registration, a CT scan was acquired after the SPECT. All images were reconstructed using vendor software (Mediso Nanoscan, Hungary) and vendor-recommended parameters.

### SPECT/CT Image Analysis

**Figure 2:**
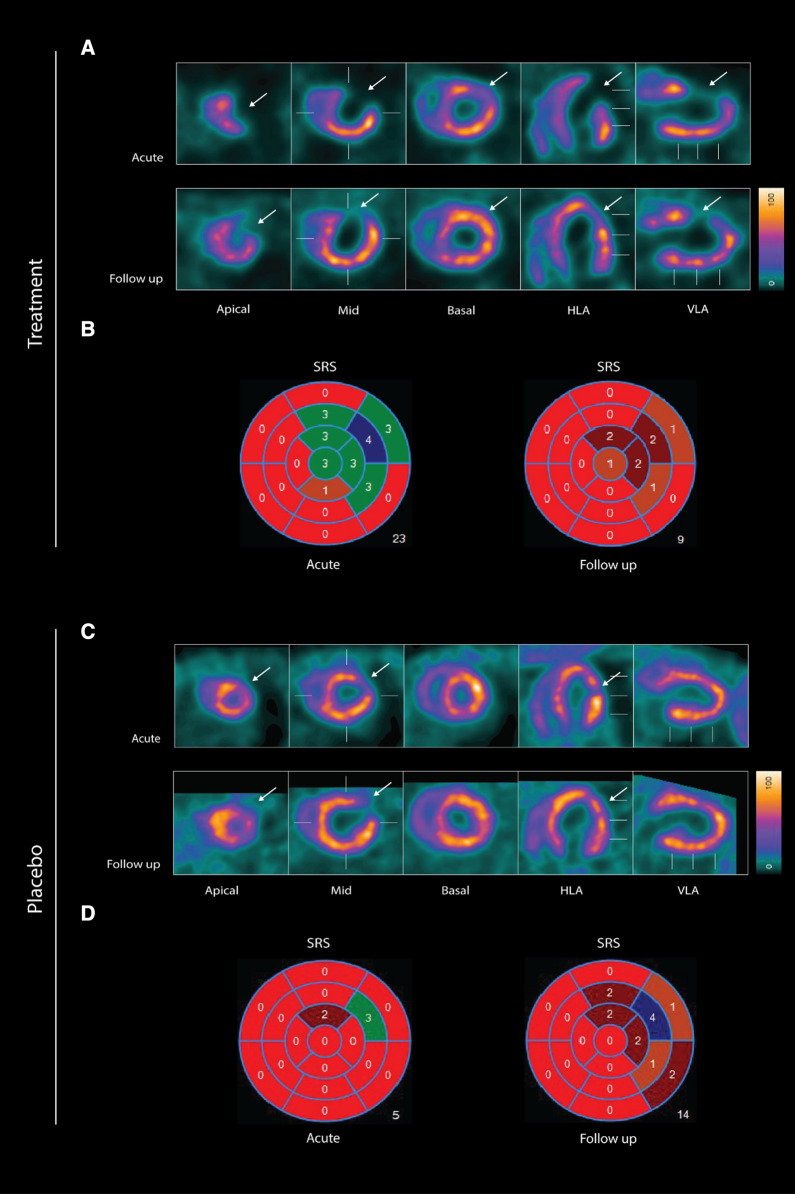
Two representative examples of rats with chronic LAD occlusion. 4DM software was used to analyze [^99m^Tc]Tc-sestamibi SPECT/CT. Top panels (**A** and **B**) show a SAX-treated rat. Bottom panels (**C** and **D**) show a placebo-treated rat. **A** Large perfusion defect in the anterior and apical wall in the acute scan, with a smaller perfusion defect at follow-up scan after SAX treatment (white arrow). **B** The perfusion defects from panel A in a 17-segment polar map. **C** Medium perfusion defect in the anterior wall at the acute scan, and a more severe perfusion defect at follow-up after placebo treatment (red arrow). **D** The perfusion defects from panel C in a 17-segment polar map. *HLA* horizontal long axis, *VLA* vertical long axis, *SRS* summed rest score

The software package Corridor4DM version 2017 (Invia LLC, USA) was used for semi-automatic analyses of the SPECT/CT data. The SPECT images were automatically oriented into short axis, vertical long axis, and horizontal long axis. The myocardial contour was then outlined automatically. Both reorientation and contouring were visually inspected and corrected by two experienced investigators in conjunction. Baseline and follow-up images were analyzed simultaneously to ensure correct and similar reorientation. The two investigators were blinded to treatment during this process. The perfusion was quantified as myocardial [^99m^Tc]Tc-sestamibi activity normalized to maximum myocardial activity, equivalent to the usual clinical standard for human subjects. Each scan was compared to a database of normal rats (developed at our site using the same species of rats and the same imaging parameters in 20 rats), to assess the perfusion, compared to a normal distribution. The perfusion parameters were obtained in myocardial segments, in accordance with the American Heart Association (AHA) 17-segment model. The perfusion in each segment was automatically graded from 0 (normal perfusion) to 4 (absent perfusion). The total perfusion score of the heart was summed from each segment as a summed rest score (SRS). The maximal SRS is 68 (4 × 17 segments) and the minimum score is 0, corresponding to a normally perfused left ventricle (Figure 2).

### Endpoint of Myocardial Recovery

Myocardial recovery was defined as the absolute difference in SRS (ΔSRS) between the acute and follow-up scans. In addition to the absolute difference in SRS, we also assessed the distribution of rats into three groups (negative, no, or positive recovery). The interscan reproducibility in infarcted Sprague-Dawley rats is on average 3.6 in SRS.^19^ Therefore, we considered a ΔSRS from − 3 to + 3 to be the normal variation and defined it as no myocardial recovery. We defined a ΔSRS ≤ -3 as negative recovery and a ΔSRS ≥ +3 as positive myocardial recovery.

### Statistics

Continuous variables are presented as medians [interquartile range, IQR]. For comparison, the non-parametric Mann–Whitney* U* test was used for unpaired data and the Wilcoxon signed-rank test was used for paired data. For associations between categorical variables, Fisher’s exact test was used. The log-rank test was used for survival analysis. For correlation analysis, Spearman’s rank test was used. A two-sided *P* value <  .05 was considered significant. All statistical analyses were performed using SPSS© version 25 (IBM SPSS, Chicago, IL, USA).

## Results

Baseline and follow-up parameters are shown in Table 1. There was a significant difference in age and weight between the two groups. The placebo group was on average six days older (*P* = .015) and weighed 75 grams more than the SAX group (*P* =  .02), with a strong correlation between age and weight (*P* < .001). There was no statistically significant difference in surgical or interventional parameters. A total of 14 rats received two sutures of the LAD artery, with no difference between the groups (*P* = .37).

**Table 1:** Baseline and follow-up characteristics of the two groups

	SAX	Placebo	*P* value
*Baseline*			
N	20	20	
Age, days	54 [49; 55]	69 [54; 69]	.015
Weight, grams	283[258; 306]	358[268; 387]	.015
Operation length, mins	28 [18; 31]	22[18; 28]	.43
Number of ligatures	1 [1; 2]	1[1; 2]	.52
Time from ligation to [^99m^Tc]Tc-injection, mins	1 [1; 3]	1 [1; 4]	.65
Time from ligation to SAX/placebo, mins	21 [20; 21]	21[20; 23]	.069
Number of rats surviving operation	16	15	
Baseline perfusion defect size, summed rest score	14[7;23]	14[5;25]	.88
*Follow-up*			
N	12	8	
Days after surgery	20[20;21]	21[21;21]	.21
Weight, grams	406[364; 423]	446 [414; 459]	.10
Follow-up perfusion defect size, summed rest score Recovery (Follow-up—baseline summed rest score)	5[2;15] 6 [.2;9]	5.5[3;11] 2 [− .5:9.5]	.61 .04

### Myocardial Recovery

The baseline left ventricular myocardium was visualized by SPECT/CT in the 31 animals that survived the first hour after myocardial infarction (SAX = 16, placebo = 15).

Median acute SRS in the entire population was 14 [7; 25], indicating a severe perfusion defect size. The acute SRSs were similar in both groups, indicating that there was no difference in size of the perfusion defect in the early stages following the LAD ligation (*P* = .88). There was no correlation between age and acute SRS (*P* = .23) or weight and acute SRS (*P* = .07).

**Figure 3:**
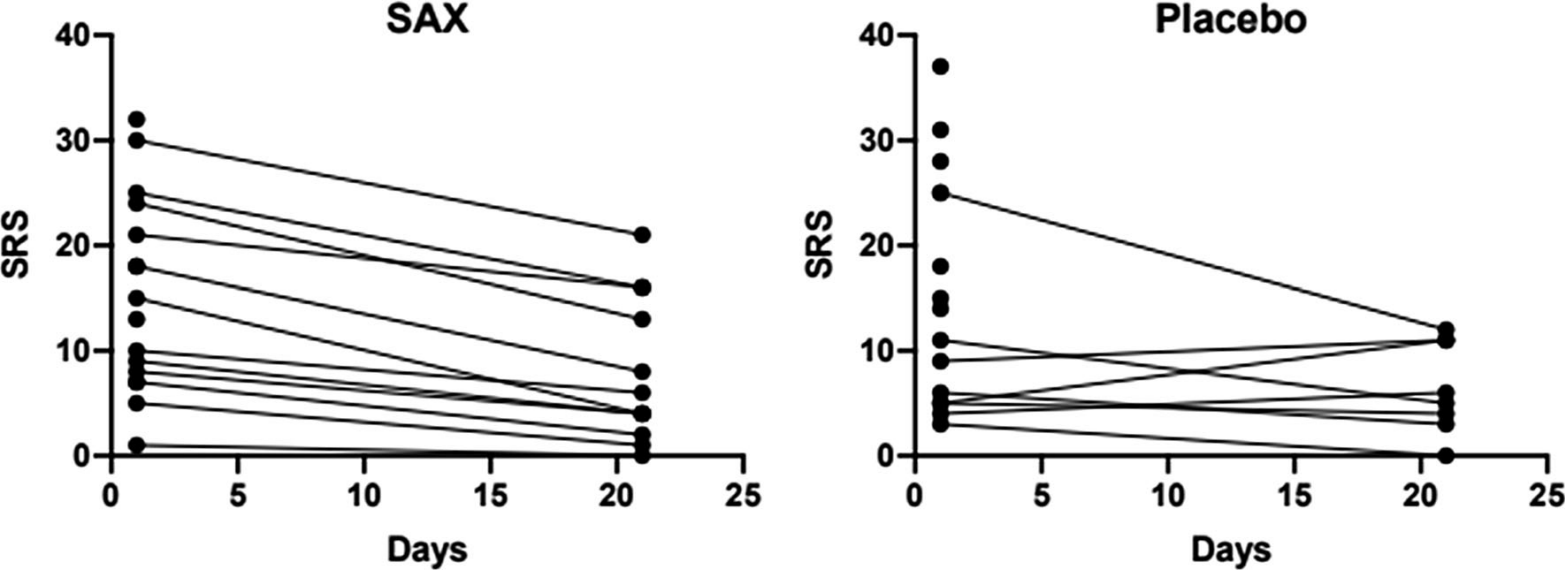
Development of SRS from acute to follow-up scan. Each line represents one rat. Rats not surviving are only shown with a dot at baseline

Twenty animals survived for three weeks and underwent follow-up SPECT/CT scan (SAX = 12, placebo = 8, Figure 3). At follow-up, the median SRS was 5.5 [3.2; 11.7], indicating a small perfusion defect size (Figure 3). The group treated with SAX had a median myocardial perfusion recovery of 6.5 [.22; 8.7] compared to only 2 [− .5; 9.5] in the placebo group, Table 1 (*P* = .04).

There was no correlation between myocardial recovery and body weight (*r* = .3, *P* = .25) or recovery and age (*r* = .2, *P* = .11).

The association between treatment and myocardial recovery remained statistically significant (*P* = .02) in a linear model controlling for the baseline infarct size (acute SRS, *P* = .01) and weight (*P* = .007), with a significant interaction between treatment and acute SRS (*P* = .02).

**Figure 4:**
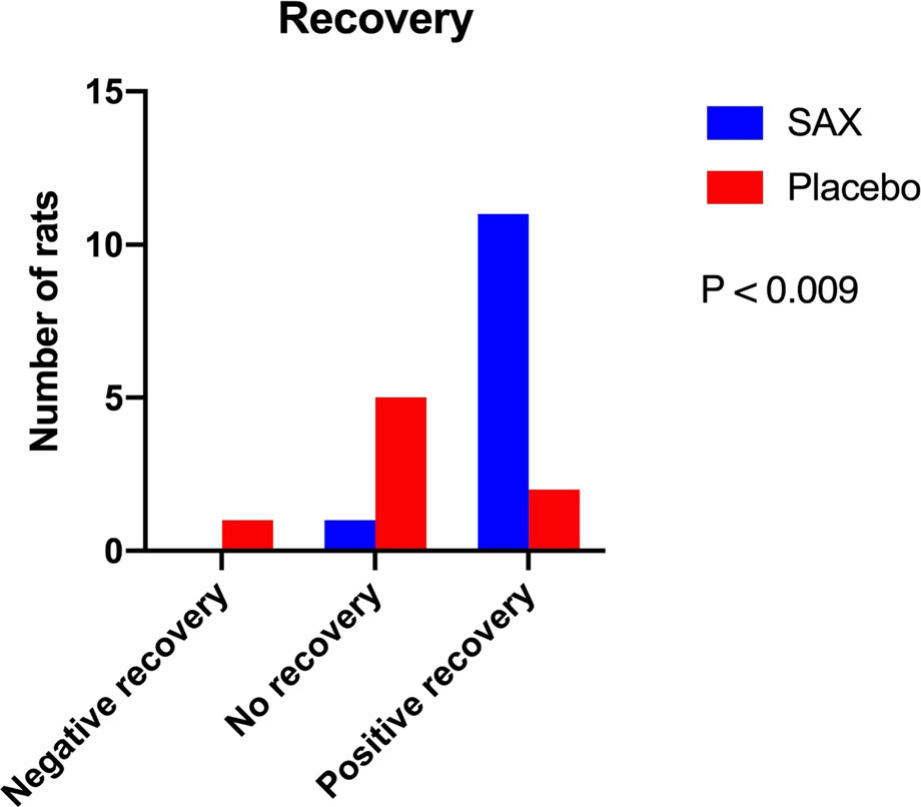
Rats divided into three groups by ΔSRS: negative recovery, no recovery, and positive recovery

The rats were further categorized into negative myocardial recovery (n = 1), no myocardial recovery (n = 6), or positive myocardial recovery (n = 13; Figure 4). This confirmed the significant difference between SAX-treated and placebo-treated animals (*P* < .009), where none of the SAX-treated animals had negative myocardial recovery and 92% of the SAX-treated animals had positive myocardial recovery.

### Mortality

**Figure 5:**
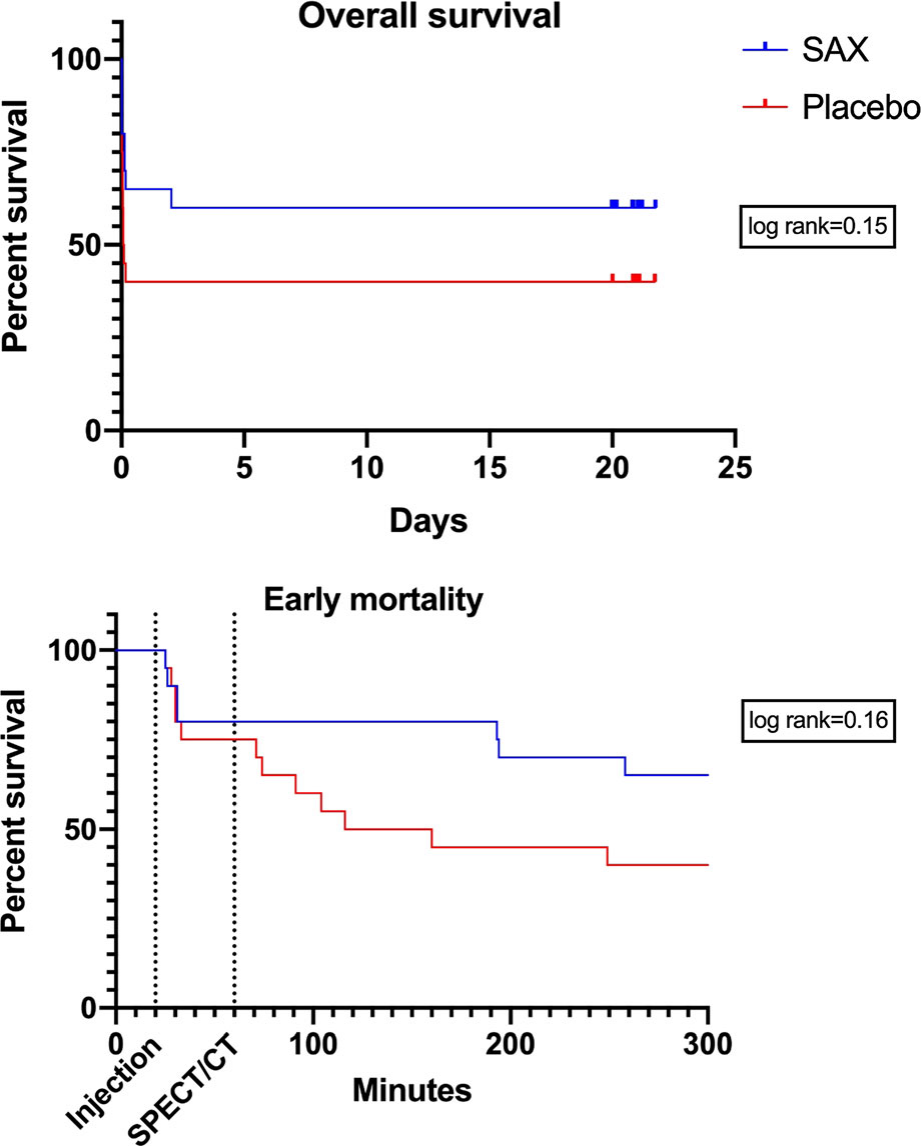
Overall survival is shown at the top. The early mortality at the bottom shows a tendency for SAX-treated rats to survive longer than the placebo-treated rats. The first dotted line is 21 min after LAD occlusion, which shows SAX or placebo injection. The second dotted line is 60 min after LAD occlusion, which shows time of SPECT/CT scan

Forty animals underwent surgery and were administered SAX or placebo. In the entire follow-up period, eight (40%) animals died in the SAX group, compared to 12 (60%) in the placebo group (Figure 5). There was a trend toward better survival in SAX-treated animals (log-rank, *P* = .15).

Nine animals died acutely, right after surgery and before SPECT/CT (SAX = 4, Placebo = 5). In these animals, there was no significant difference in median time to death from end of operation, between animals treated with SAX (9 min [6; 10]) and placebo (5 min [2; 8]) (*P* = 1.0). Eleven animals died after baseline SPECT/CT (SAX = 4, Placebo = 7). In these 11 animals, there was no statistically significant difference in median acute SRS between SAX and placebo (15.5 [8.5; 28.5] and 25 [15; 31], P = .34), but we observed a significant difference in median time to death from end of operation between animals treated with SAX (206 min [172; 2248]) and placebo (82 min [53; 141]) (*P* = .024).

## Discussion

Three weeks after myocardial infarction with permanent LAD occlusion, a defect in myocardial perfusion was evident, indicating a successful model of myocardial infarction. The group treated with SAX had a significantly better myocardial recovery of perfusion compared to the placebo group, suggesting that this novel CGRP analogue improves myocardial recovery after myocardial infarction. Our study indicates that SAX decreases mortality using this model for myocardial infarction.

### SAX

SAX is a vasodilatory CGRP analogue that acts on various arteries with a tenfold lower potency than native CGRP in all situations analyzed so far.^14^,^16^ In our infarct model, most of the myocardium normally supplied by the occluded LAD will obviously not benefit from a vasodilator. However, at the border of the territory, there will be some supply from “neighboring” non-occluded arteries and, although not addressed in this study, the vasodilatory effect in this non-occluded myocardial vasculature may be hypothesized as part of the mode-of-action of the observed perfusion recovery. Further, in addition to vasodilation, CGRP is known to induce endothelial proliferation and angiogenesis and, given that the SAX-induced recovery of myocardial perfusion is measured three weeks after occlusion and SAX treatment, angiogenesis could also be the mode-of-action.^7^

A concern regarding SAX and other systemic vasodilator treatments of myocardial ischemia is a coronary steal effect of the coronary arteries 20 in the presence of a flow that limits coronary stenosis. When treating with a vasodilating drug, the vessels in the non-ischemic area dilate and this could divert coronary flow from the ischemic area to the non-ischemic area. This potential effect could therefore reduce the perfusion to the ischemic area. However, since our infarct model was a permanent total occlusion, we would not expect to see any coronary steal.

### Myocardial Infarction

The most common method of inducing myocardial infarction in animal models is with ligation of the LAD coronary artery. This method has been used since it was first described by Johns and Olson in 1954.^21^ However, the final infarct size has a large variation ranging from 4 to 59%. This is because of inconsistency in the ligation during surgery. In our study, the variation in infarct size was between 10% and 37%. This variation in infarct size could be because of the placement of ligature or number of ligatures. Srikanth et al. placed the ligature, 8 mm, from the origin of the LAD. This placement seems to give a minor variation in infarct size, but does not induce large infarction, with a reported infarct size variation from 17% to 25%.^22^ We placed the ligature more proximal, around 5 mm from the origin, to ensure larger infarct size. Despite the fact that our data show a large variation in infarct size, there was no difference in size of the perfusion defect size before first SAX/placebo treatment between the two groups.

This myocardial infarction model has a high early mortality rate. John et al. 21 showed that, in a chronic occlusion model, the surgical mortality was 21% and late death was 12%. In our study, the surgical mortality was 23%, which was expected. We had a late mortality of 28%, which is higher than that seen in other studies. A higher incidence of myocardial infarction could explain this higher mortality. In our study, 100% of the animals had an infarction, compared to only 83% of the animals in the study by John et al.^21^

### Recovery

SAX showed a significant improvement in perfusion recovery compared to placebo. In the group treated with SAX, there was a median reduction in SRS of 6 points (corresponding to a mean relative reduction of 55%), compared to only 2 points (corresponding to a mean relative reduction of only 11%) in the placebo group. Chronic occlusion of the coronary artery is used in our study; therefore, recovery is not as great as that seen in studies in which a reperfusion infarct model was used. Pfeffer et al. described the development in infarct size in rats with chronic occlusion. The infarct size was 41% after 1 day and 39% after day 106.^23^ The myocardial infarction was verified using histology and therefore not comparable to our study. However, it shows that the infarct size does not reduce significantly for 106 days when there is no intervention. Therefore, our reduction in final infarct size shows a promising cardioprotective aspect of SAX.

In the placebo group, a majority of rats had negative or no recovery, while none in the SAX-treated group had a negative recovery. An explanation for this could be the physiological effects of SAX regarding the dilation of the epicardial coronary arteries.^4^

A strength of the model used for determining recovery is the radiopharmaceutical properties of [^99m^Tc]Tc-sestamibi. [^99m^Tc]Tc-sestamibi enters the myocytes easily and accumulates in the mitochondria, because it cannot metabolize further, and redistribution is minimal.^24^ The injection of [^99m^Tc]Tc-sestamibi in our study was done 20 min before administering SAX or placebo; therefore, the effects of the pharmacological treatment do not affect distribution of the radiotracer. The acute distribution of [^99m^Tc]Tc-sestamibi shows the myocardial area at risk of infarction, and the follow-up SPECT/CT three weeks after intervention shows the final infarct.

### In Vivo Quantification Using SPECT

The use of [^99m^Tc]Tc-sestamibi in rats is well established. The half-life, the extraction in the myocardium, and no large positron range are advantages when examining small rodents.^18^ Conversely, [^99m^Tc]Tc-sestamibi is not the optimal choice in humans when examining myocardial blood flow, because of the long half-life and gamma decay. ^82^Rubidium is a short-lived positron emission tomography (PET) tracer, used to detect perfusion defects and assess myocardial blood flow. However, the positron range is large and therefore it is hard to discriminate between infarcted and healthy myocardium in small animals. A novel PET tracer, [^18^F]-flurpiridaz, is promising in assessing perfusion defects and myocardial blood flow.^25^ The positron range is low, which enables its use in small rodents, with great contrast between infarcted and healthy myocardium. However, so far [^18^F]-flurpiridaz is not commercially available and was thus not usable in our study.

### Survival

There was no significant difference in overall survival between the two groups, although there was a tendency toward a higher survival rate in the SAX-treated group. This tendency suggests that SAX does not have a damaging effect on the animals or the myocardium.

In the rats that died before SPECT/CT scan, there was no difference between the two groups regarding treatment with SAX or placebo, or the length of survival. Nilsson et al. have shown that the effect of SAX is most pronounced three hours after treatment, which means that the cardioprotective effect of SAX has not yet been established in rats that had died before the SPECT/CT scan.^14^ This corresponds to our finding that time to death was similar in the acute phase (first hour after infarction).

In the rats that died in the subacute phase (after a potential effect of SAX), we observed that the SAX group survived significantly longer than the placebo group, which supports the data suggesting a cardioprotective effect of SAX.

### Limitations

In the present study, SAX was the only drug tested and was not compared to other CGRP analogues or other types of vasodilating drugs. There was a significant difference in age and weight between the two groups. The mean difference in age was six days, but we found no indication that this difference affected acute SRS or recovery.

In this study, we did not confirm the infarct size measured with SPECT to either histology or triphenyltetrazolium chloride (TTC) staining. However, the correlation between infarct size measured by [^99m^Tc]Tc-sestamibi and TTC-staining, in a model with permanent ligation of the LAD, has been showed in Sprague-Dawley rats.^26^

In this study, a chronic occlusion of the coronary artery was made to evaluate the potential cardioprotective effects of SAX with no other kind of intervention. A more clinically relevant model would be a reperfusion model, where the coronary artery is occluded for a limited amount of time, mimicking patients undergoing PCI. However, the reperfusion model shows only small perfusion defect size and the potential myocardial improvement by SAX would be harder to determine.

## New Knowledge Gained

An analogue of CGRP that induces both coronary and peripheral vasodilation significantly improves myocardial perfusion recovery after experimental myocardial infarction in rats. There was no significant difference in overall survival between the two groups suggesting that SAX does not have a damaging effect on the animals or the myocardium.

## Conclusion

In conclusion, the CGRP analogue, SAX, seems to have a cardioprotective effect on a rat model of myocardial infarction, by improving the perfusion recovery after a chronic occlusion of the coronary artery.

## Supplementary Information

Below is the link to the electronic supplementary material.

Electronic supplementary material 1 (PPTX 3168 kb)

## Abbreviations

MI: Myocardial infarction
CGRP: Calcitonin gene-related peptide
PCI: Percutaneous coronary intervention
LAD: Left anterior descending coronary artery
SPECT: Single photon emission computed tomography
CT: Computed tomography
ECG: Electrocardiogram
SRS: Summed rest score
PET: Positron emission tomography

## References

[1] Cahill TJ, Kharbanda RK (2017). Heart failure after myocardial infarction in the era of primary percutaneous coronary intervention: mechanisms, incidence and identification of patients at risk. World J Cardiol..

[2] O’Connor RE, Brady W, Brooks SC, Diercks D, Egan J, Ghaemmaghami C, et al. Part 10: Acute coronary syndromes: 2010 American Heart Association Guidelines for Cardiopulmonary Resuscitation and Emergency Cardiovascular Care., Circulation 2010;122. 10.1161/CIRCULATIONAHA.110.971028 20956226

[3] Brain SD, Williams TJ, Tippins JR, Morris HR, MacIntyre I (1985). Calcitonin gene-related peptide is a potent vasodilator. Nature.

[4] Russell FA, King R, Smillie SJ, Kodji X, Brain SD (2014). Calcitonin gene-related peptide: physiology and pathophysiology. Physiol Rev.

[5] Sheykhzade M, Amandi N, Pla MV, Abdolalizadeh B, Sams A, Warfvinge K (2017). Binding and functional pharmacological characteristics of gepant-type antagonists in rat brain and mesenteric arteries. Vascul Pharmacol.

[6] Prieto D, Benedito S, Nyborg NC (1991). Heterogeneous involvement of endothelium in calcitonin gene-related peptide-induced relaxation in coronary arteries from rat. Br J Pharmacol.

[7] Sohn I, Sheykhzade M, Edvinsson L, Sams A. The effects of CGRP in vascular tissue - Classical vasodilation, shadowed effects and systemic dilemmas. European Journal of Pharmacology 2020: Vol. 881. 10.1016/j.ejphar.2020.173205 32442540

[8] Chai W, Mehrotra S, Jan Danser AH, Schoemaker RG (2006). The role of calcitonin gene-related peptide (CGRP) in ischemic preconditioning in isolated rat hearts. Eur J Pharmacol.

[9] Sun X-J, Pan S-S (2014). Role of calcitonin gene-related peptide in cardioprotection of short-term and long-term exercise preconditioning. J Cardiovasc Pharmacol.

[10] Franco-Cereceda A, Lundberg JM (1985). Calcitonin gene-related peptide (CGRP) and capsaicin-induced stimulation of heart contractile rate and force. Naunyn Schmiedebergs Arch Pharmacol..

[11] Johnston FG, Bell BA, Robertson IJA, Miller JD, Haliburn C, O’Shaughnessy D (1990). Effect of calcitonin-gene-related peptide on postoperative neurological deficits after subarachnoid haemorrhage. Lancet.

[12] Effect of calcitonin-gene-related peptide in patients with delayed postoperative cerebral ischaemia after aneurysmal subarachnoid haemorrhage. European CGRP in Subarachnoid Haemorrhage Study Group. Lancet (London, England) 1992;339:831–4. 1347857

[13] Juul R, Aakhus S, Björnstad K, Gisvold SE, Brubakk AO, Edvinsson L (1994). Calcitonin gene-related peptide (human alpha-CGRP) counteracts vasoconstriction in human subarachnoid haemorrhage. Neurosci Lett.

[14] Nilsson C, Hansen TK, Rosenquist C, Hartmann B, Kodra JT, Lau JF (2016). Long acting analogue of the calcitonin gene-related peptide induces positive metabolic effects and secretion of the glucagon-like peptide-1. Eur J Pharmacol.

[15] Aubdool AA, Thakore P, Argunhan F, Smillie SJ, Schnelle M, Srivastava S (2017). A novel α-calcitonin gene-related peptide analogue protects against end-organ damage in experimental hypertension, cardiac hypertrophy, and heart failure. Circulation.

[16] Sheykhzade M, Abdolalizadeh B, Koole C, Pickering DS, Dreisig K, Johansson SE (2018). Vascular and molecular pharmacology of the metabolically stable CGRP analogue. SAX. Eur J Pharmacol..

[17] Hatada K, Riou LM, Ruiz M, Yamamichi Y, Duatti A, Lima RL (2004). 99mTc-N-DBODC5, a new myocardial perfusion imaging agent with rapid liver clearance: comparison with 99mTc-sestamibi and 99mTc-tetrofosmin in rats. J Nucl Med.

[18] Liu Z, Kastis GA, Stevenson GD, Barrett HH, Furenlid LR, Kupinski MA (2002). Quantitative analysis of acute myocardial infarct in rat hearts with ischemia-reperfusion using a high-resolution stationary SPECT system. J Nucl Med.

[19] Strydhorst JH, Leenen FH, Ruddy TD, Wells RG (2011). Reproducibility of serial left ventricle perfusion, volume, and ejection fraction measurements using multiplexed multipinhole SPECT in healthy rats and rats after myocardial infarction. J Nucl Med.

[20] Becker LC (1978). Conditions for vasodilator-induced coronary steal in experimental myocardial ischemia. Circulation.

[21] Johns TNP, Olson BJ (1954). Experimental myocardial infarction. I. A method of coronary occlusion in small animals. Ann. Surg..

[22] Srikanth G, Prakash P, Tripathy N, Dikshit M, Nityanand S (2009). Establishment of a rat model of myocardial infarction with a high survival rate: a suitable model for evaluation of efficacy of stem cell therapy. J Stem Cells Regen Med..

[23] Pfeffer JM, Pfeffer MA, Fletcher PJ, Braunwald E (1991). Progressive ventricular remodeling in rat with myocardial infarction. Am J Physiol.

[24] Beller GA, Sinusas AJ. Experimental studies of the physiologic properties of technetium-99m isonitriles. Am J Cardiol 1990;66(13). 10.1016/0002-9149(90)90605-z 2145747

[25] Maddahi J, Lazewatsky J, Udelson J, Berman D, Beanlands R, Heller G et al. Phase-III clinical trial of fluorine-18 flurpiridaz positron emission tomography for evaluation of coronary artery disease. J Am Coll Cardiol 2020;76. 10.1016/j.jacc.2020.05.063 32703509

[26] Acton P, Thomas D, Zhou R (2006). Quantitative imaging of myocardial infarcts in rats with high resolution pinhole SPECT. Int J Cardiovasc Imaging.

