# Supplementary material for: Myocardial perfusion recovery induced by an α-calcitonin gene-related peptide analogue
Source: J Nucl Cardiol. 2021 Jun 4;29(5):2090–9. doi: 10.1007/s12350-021-02678-8 (PMC9553834; doi:10.1007/s12350-021-02678-8)
Supplement: Supplementary file 1 — Electronic supplementary material 1 (PPTX 3168 kb) [file 12350_2021_2678_MOESM1_ESM.pptx]

## Slide 1
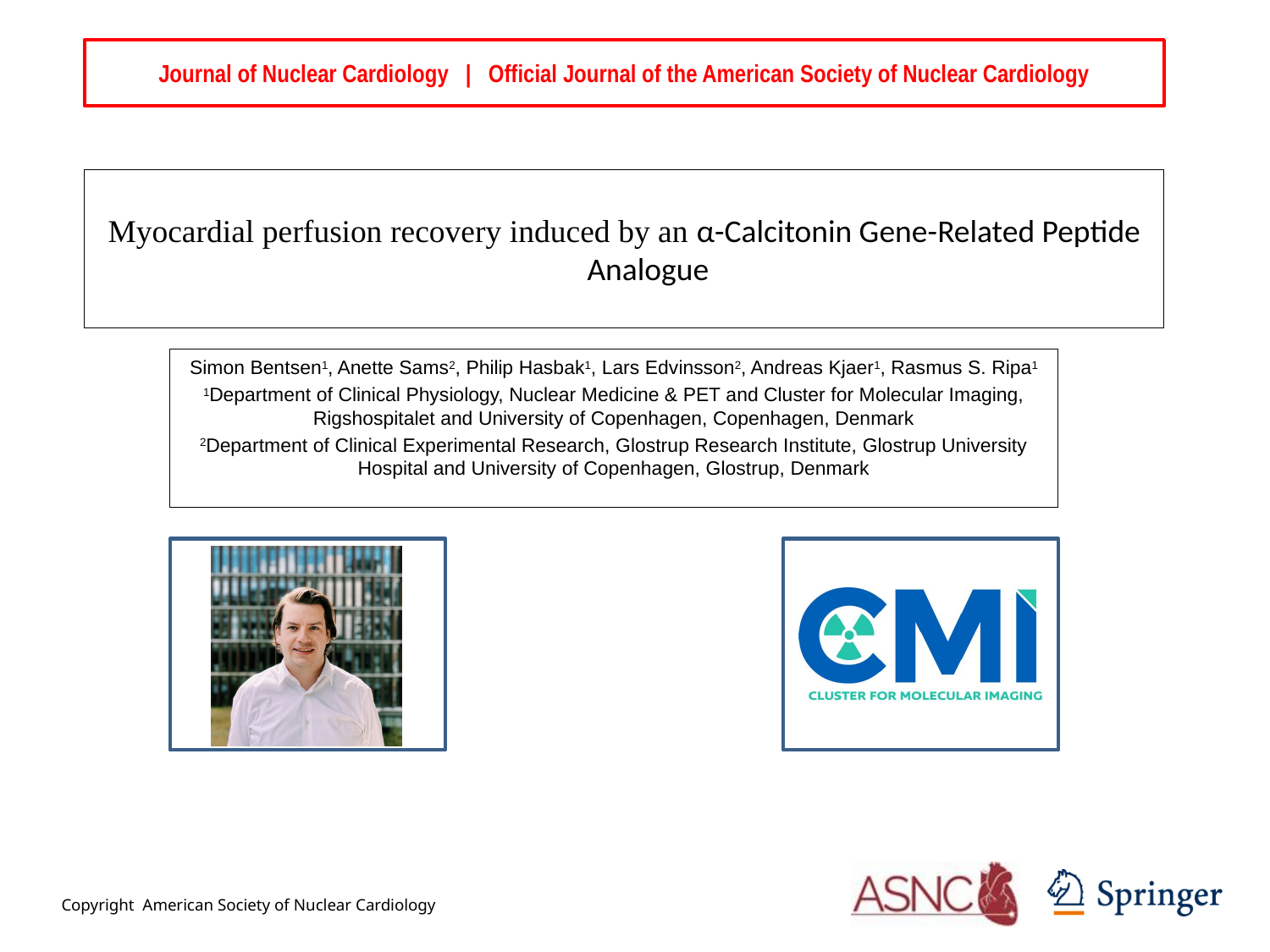

Journal of Nuclear Cardiology | Official Journal of the American Society of Nuclear Cardiology
# Myocardial perfusion recovery induced by an α-Calcitonin Gene-Related Peptide Analogue
Simon Bentsen1, Anette Sams2, Philip Hasbak1, Lars Edvinsson2, Andreas Kjaer1, Rasmus S. Ripa1
1Department of Clinical Physiology, Nuclear Medicine & PET and Cluster for Molecular Imaging, Rigshospitalet and University of Copenhagen, Copenhagen, Denmark
2Department of Clinical Experimental Research, Glostrup Research Institute, Glostrup University Hospital and University of Copenhagen, Glostrup, Denmark
Copyright American Society of Nuclear Cardiology

## Slide 2
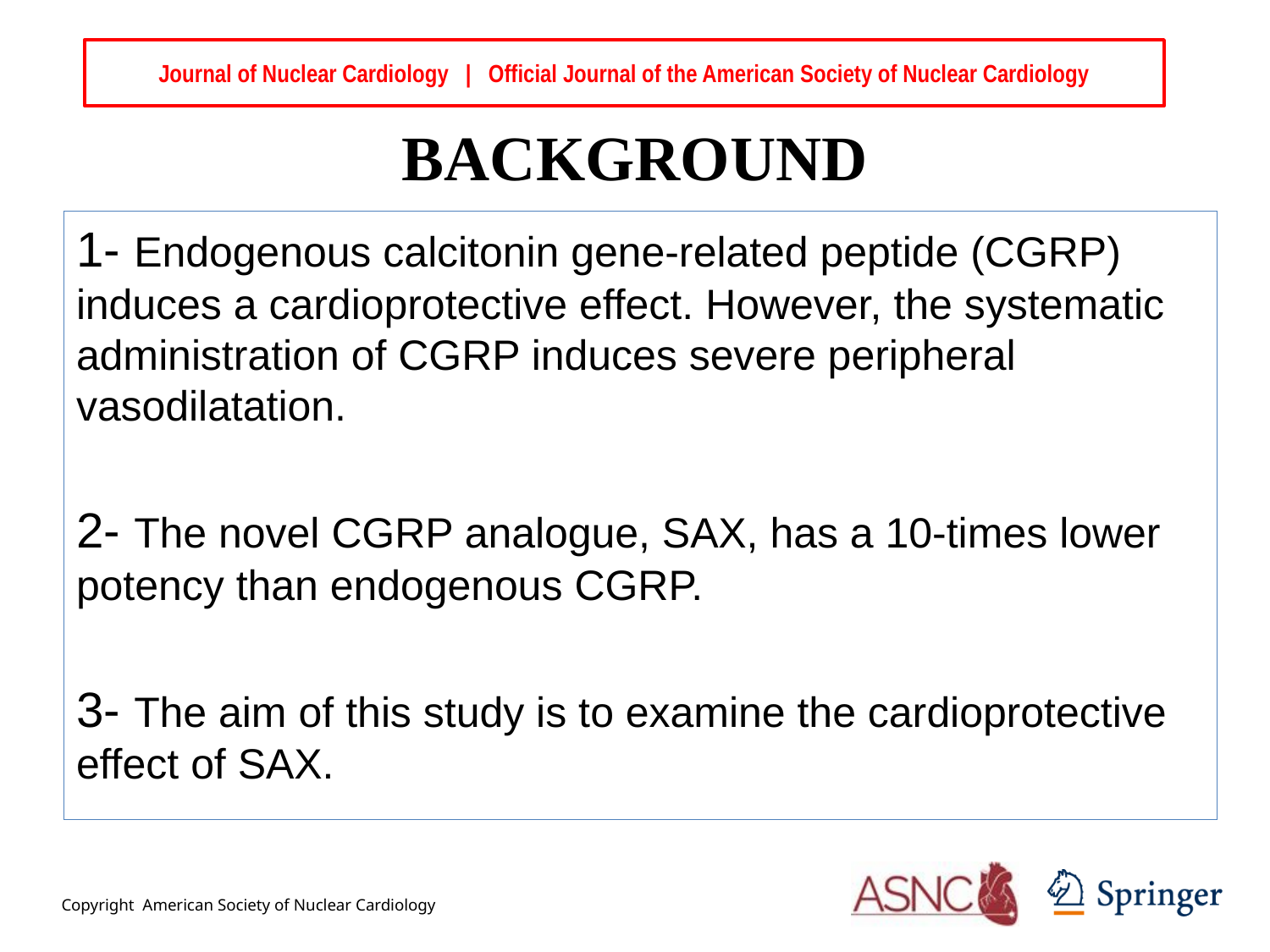

Journal of Nuclear Cardiology | Official Journal of the American Society of Nuclear Cardiology
# BACKGROUND
1- Endogenous calcitonin gene-related peptide (CGRP) induces a cardioprotective effect. However, the systematic administration of CGRP induces severe peripheral vasodilatation.
2- The novel CGRP analogue, SAX, has a 10-times lower potency than endogenous CGRP.
3- The aim of this study is to examine the cardioprotective effect of SAX.
Copyright American Society of Nuclear Cardiology

## Slide 3
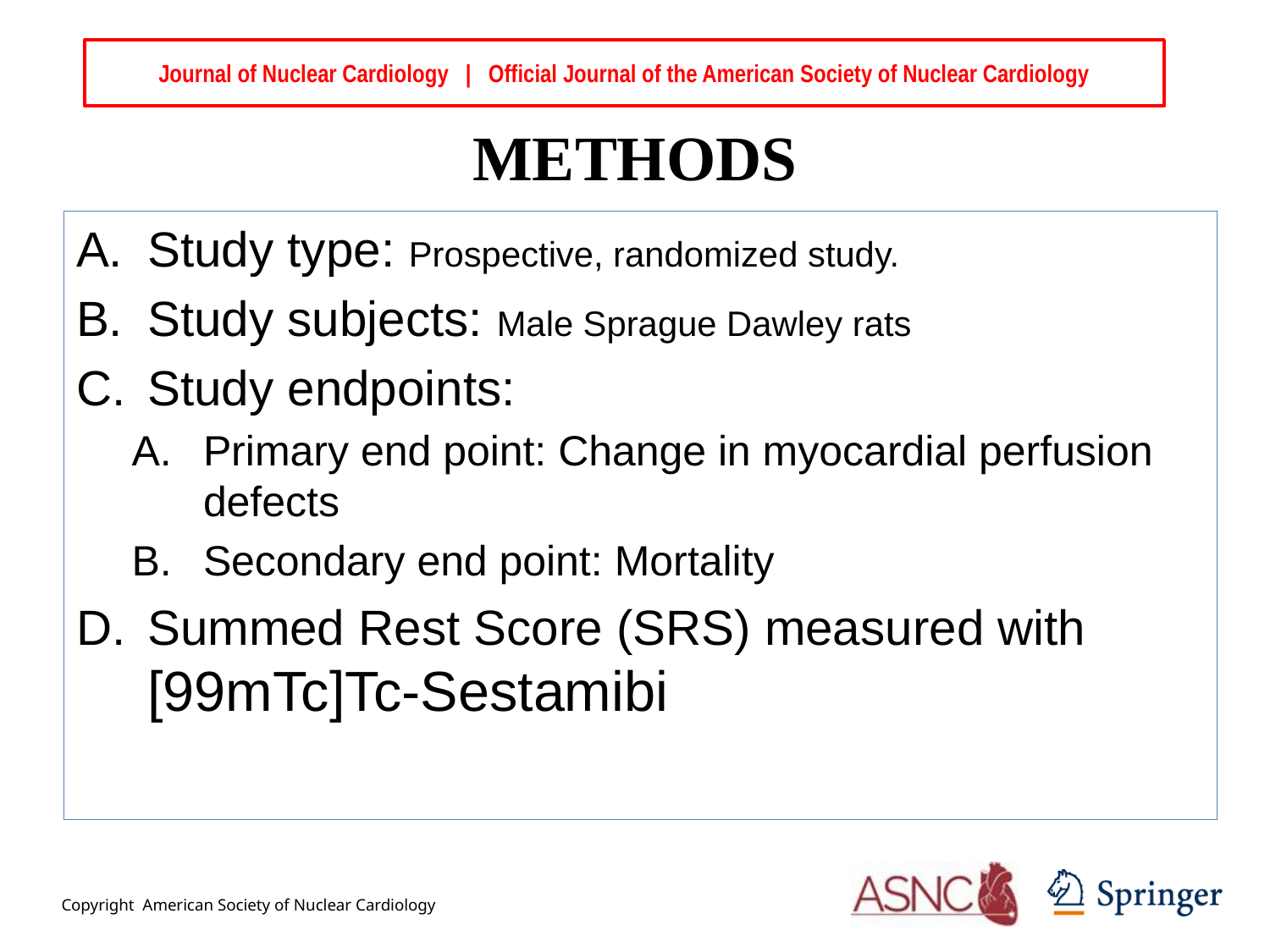

Journal of Nuclear Cardiology | Official Journal of the American Society of Nuclear Cardiology
# METHODS
Study type: Prospective, randomized study.
Study subjects: Male Sprague Dawley rats
Study endpoints:
Primary end point: Change in myocardial perfusion defects
Secondary end point: Mortality
Summed Rest Score (SRS) measured with [99mTc]Tc-Sestamibi
Copyright American Society of Nuclear Cardiology

## Slide 4
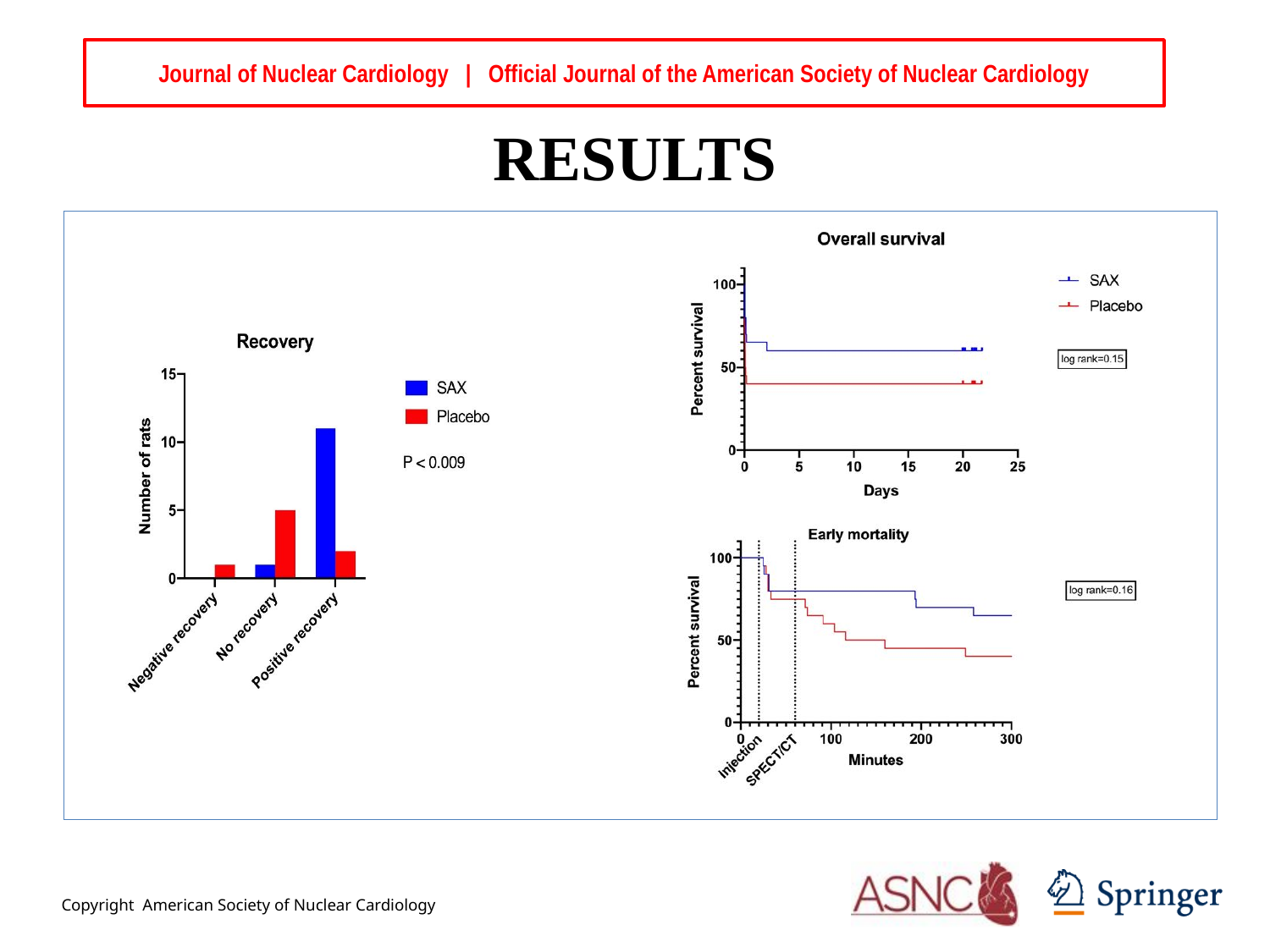

Journal of Nuclear Cardiology | Official Journal of the American Society of Nuclear Cardiology
# RESULTS
Copyright American Society of Nuclear Cardiology

## Slide 5
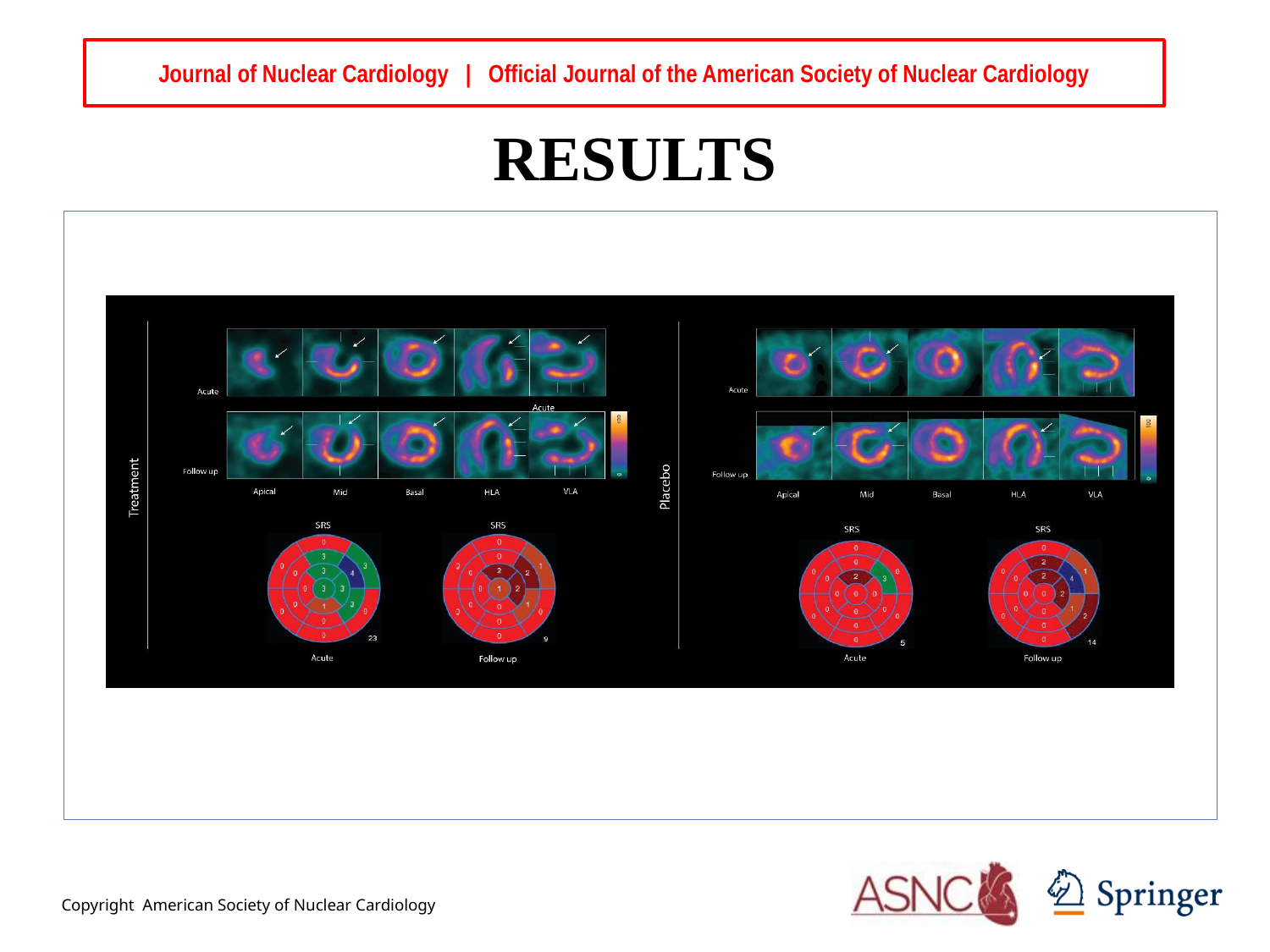

Journal of Nuclear Cardiology | Official Journal of the American Society of Nuclear Cardiology
# RESULTS
Copyright American Society of Nuclear Cardiology

## Slide 6
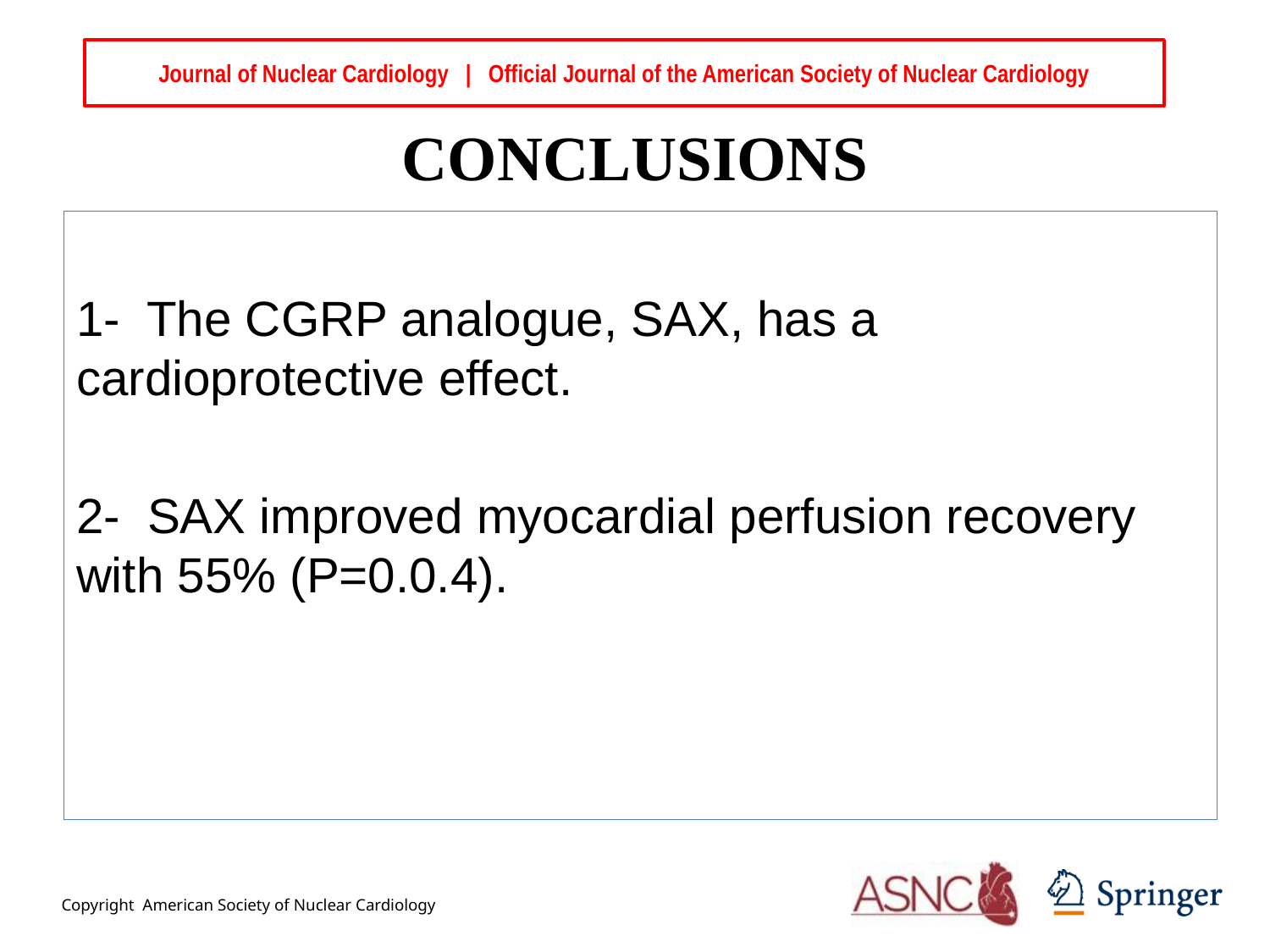

Journal of Nuclear Cardiology | Official Journal of the American Society of Nuclear Cardiology
# CONCLUSIONS
1- The CGRP analogue, SAX, has a cardioprotective effect.
2- SAX improved myocardial perfusion recovery with 55% (P=0.0.4).
Copyright American Society of Nuclear Cardiology
